# Rapid Prediction and Accurate Location Selection of Mild Traumatic Brain Injury (mTBI) by Using Multiple Parameter Analysis of Diffusion Tensor Imaging (DTI): Integrating Correlational and Clinical Approaches

**DOI:** 10.1155/2023/7467479

**Published:** 2023-01-16

**Authors:** Wei Wei, Na Li, Xianping Du, Zhongyi Sun, Weiliang Chen, Pengfei Rong, Jian Shi

**Affiliations:** ^1^Department of Radiology, The Third Xiangya Hospital, Central South University, 410013 Changsha, China; ^2^Department of Mechanical and Aerospace Engineering, Rutgers University, 08854 Piscataway, USA; ^3^Department of Neurosurgery, Xiangya Hospital, Central South University, 410018 Changsha, China; ^4^Department of Neurosurgery, Haining People's Hospital, 314499 Zhejiang, China; ^5^Department of Hematology and Critical Care Medicine, The Third Xiangya Hospital, Central South University, Changsha, China

## Abstract

**Background:**

Mild traumatic brain injury (mTBI) is a widespread and serious public health problem which also causes physical and psychological suffering to patients and their families and imposes a significant economic burden on society. But it is usually very difficult to detect and provide warning of mTBI in early stage. Therefore, a novel method is urgent for the increasing demands on the accurate and rapid prediction and feature selection of mTBI.

**Objectives:**

To establish a better idea of the performance of neuroimage biomarker in the acute phase of mTBI, our study adopts diffusion tensor imaging (DTI) which could present the pathophysiological changes of white matter through several parameters noninvasively and combined with behavioral experiments such as intelligence quotient test, memory, executive function, and motion function to find the relationship between DTI abnormal brain regions and behavioral abnormalities. Then, provide new method for rapid prediction and feature selection of mTBI.

**Methods:**

77 mTBI patients were admitted to the Emergency and Neurosurgery Departments of the Third Xiangya Hospital of Central South University from August 2019 to July 2021; the patients (41 males and 36 females) suffered mTBI because of car accident (36), assault (11), and fall (30). All the mTBI patients were examined through MRI scan and behavioral psychology test within 3 days after injury. MRI images and behavioral psychology tests were also collected; the correlation between the DTI biomarker and the cognitive psychological outcome was analyzed. A series of integration and computational methods were also used for fusion arithmetic and result analysis.

**Results:**

Compared with the healthy control group, the patients in the acute stage of mTBI presented lower scores in the digit symbol substitution test (DSST), suggesting that mTBI patients in the acute stage had decline in information processing speed and associative learning. The difference of DTI parameters in acute stage mTBI patients was mainly manifested as increased AD and MD values in multiple brain regions, while RD and FA values have no significant difference. The most significant brain regions were bilateral corticospinal tracts (CST), bilateral posterior internal capsule lentiform nucleus, bilateral superior longitudinal fasciculus, left terminal striae, and left sagittal plane with right posterior thalamic radiation. The Pearson correlation coefficient was significantly positive correlation between AD and MD elevation in the left sagittal layer and the results of DSST and digit span in acute stage mTBI patients.

**Conclusions:**

The acute phase mTBI patients performed lower score on the DSST than those in the normal control group. This neuropsychological change was associated with increased AD value and MD value in the left sagittal layer, which indicated reduction of information processing speed in mTBI patients in the acute phase. It might be related to abnormal AD value and MD value in the upper longitudinal tract, lower longitudinal tract, lower frontal occipital tract, and sagittal layer. In this study, combined with neuropsychological test and increase of the AD value and MD value in certain brain region, neurosurgeon should pay more attention to the abnormal of the upper longitudinal tract and the patients' information processing speed in the diagnosis and treatment of the acute phase mTBI patients. The study offers a much more secure and integrated method for rapid prediction and feature selection of mTBI, which could have broader clinical approaches and application prospects.

## 1. Introduction

Mild traumatic brain injury (mTBI) is the most frequent traumatic brain injury in clinical neurosurgery. Most mTBI patients present normally in CT and MRI scans; hence, both doctors and patients do not pay enough attention to this *silent* disease, which easily leads to mTBI patients missing the optimal intervention period [1]. mTBI patients may suffer long-term physical, cognitive, and behavioral symptoms, including headache, memory and attention deficit, abnormal executive function, dizziness, anxiety, depression, insomnia, fatigue, irritability, and other manifestations [2]. Among mTBI patients, about 20-30% are subjective to these symptoms for 3 months or even several years, known as postconcussion syndrome (PCS) [3].

Pathological examinations in TBI animals and humans often report axon *bubble effect* of mTBI swelling in the acute stage [4], subacute, and chronic phase. The main pathology is demyelinating lesions when the axon is stretched in mechanical stimulation [5]: the stretched axon leads to higher permeability of calcium ion channels causing the inflow Ca2 +; up to a certain level of Ca2+, the Ca2+ proteinases are activated while the cytoskeleton of neurons and axons is decomposed, finally leading to lysis of the axon cytoskeleton and demyelination. Most researchers tend to believe that repairable axonal swelling is more likely to occur than unrepairable axonal rupture in mTBI acute stage [6]. However, the correlation between acute mTBI and clinical symptoms is still not clear.

Diffusion tensor imaging (DTI) can noninvasively present the pathophysiological changes of white matter through the mean diffusivity (MD), fractional anisotropy (FA), and axial diffusivity (AD) [7]. At present, researchers mainly focus on the subacute and chronic phases of mTBI, with few research on the acute phase. Previous studies focused more on the fMRI in mTBI subacute and mTBI chronic stages, and in recent years, more researchers have compared the difference in fMRI parameters between the acute and chronic stages [8]. The FA values of the mTBI patients in the subacute stage were found lower than the control group in the corpus callosum, internal capsule, external capsule, corona radiata, and superior longitudinal fasciculus [9]. Lo et al. [10] found that the left corpus callosum FA values in the left corpus callosum were decreased in the mTBI patients 2 years after injury. Greenberg et al. [11] found lower FA values in chronic mTBI patients in the bilateral deep fronto-temporal and bilateral corpus callosum decreased in chronic mTBI patients. Ljungqvist et al. [5] reported that FA values of the corpus callosum in chronic mTBI patients (more than 6 months after injury) decreased and RD values increased compared with the control group. Recently, researchers tend to pay more attention to the different manifestations of acute and chronic mTBI. Palacios et al. [8] found that mTBI patients had different manifestations in various DTI parameters 2 weeks and 6 months after injuries in their longitudinal study of white matter microstructural changes in DTI.

To better understand the performance of neuroimage biomarkers of DTI in the acute phase of mTBI, our study adopts both DTI and behavioral experiments including intelligence quotient (IQ), memory, executive function, and motion function: FA, AD, and MD values of the mTBI group and healthy control group were, respectively, calculated; then, the correlation analysis was implemented between behavioral abnormalities and DTI parameters which were found significantly higher in the brain regions; finally, the relationship between DTI abnormal brain regions and behavioral abnormalities was obtained.

## 2. Method and Material

**Figure 1:**
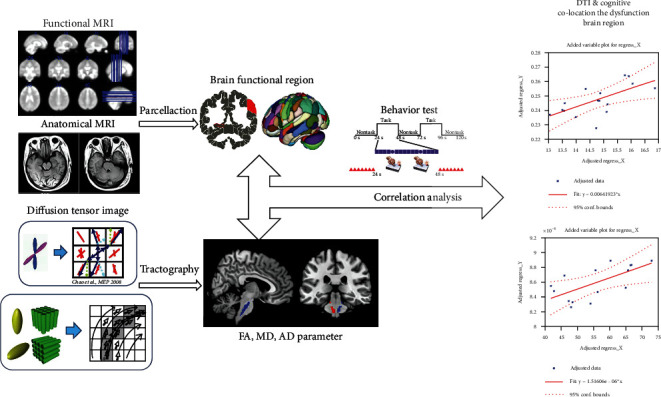
Overview of image analysis process.

A total of 77 mTBI patients were admitted to the Emergency and Neurosurgery Departments of the Third Xiangya Hospital of Central South University from August 2019 to July 2021. The patients (41 males and 36 females) suffered mTBI because of car accidents (36), assaults (11), and falls (30). The main design of the experiment is shown in Figure 1. All the mTBI patients were examined through MRI scan and behavioral psychology test 3 days after injuries. MRI images and behavioral psychology tests were also collected. The correlation between the DTI biomarker and the cognitive psychological outcome was analyzed. The control group consisted of 72 healthy subjects matched with the mTBI group in terms of gender, age, and education level. Both the mTBI patients and the control group signed the informed consent with the ethical approval documents approved by the ethics committee of Third Xiangya Hospital, Central South University. The inclusion and exclusion criteria were the same as those of our previous mTBI studies, as shown in the supplementary material.

### 2.1. Neuropsychological Assessment

To measure the cognitive level of the mTBI patients in the acute stage, the patient and control groups were examined with 14 cognitive behavioral tests, including IQ, general knowledge, digit span, mapping, digit symbols, and stroop test. These behavioral tests can reflect the patients' memory, attention, executive functions, learning association ability, visual memory, and logical association ability.

### 2.2. Image Acquisition

Both the patient and control groups underwent the same MRI protocol using the 8-channel head orthogonal coil of Philips INGENIA 3.0T MRI (Philips Co., Holland) in Third Xiangya Hospital, Central South University. The patient group was examined within 3 days after mTBI injuries (the same day as the neuropsychological examination). All subjects were placed on the scanning bed with their heads fixed while keeping their heads as still as possible, staying awake, and not thinking anything or processing any tasks during the examination. The specific scanning parameters were the same as those in our previous mTBI studies, as shown in the supplementary materials [1].

### 2.3. MRI Image Processing and Analysis

A diffusion tensor *D* is used in the DTI to describe the movements of molecules in all directions, including the combined movements in different directions. The matrix *D* is a symmetric 2nd order tensor, as shown in

(1) $$\begin{array}{c} D=\left[\begin{array}{ccc} D_{xx} & D_{xy} & D_{xz}\\ D_{yx} & D_{yy} & D_{yz}\\ D_{zx} & D_{zy} & D_{zz} \end{array}\right]. \end{array}$$

**Figure 2:**
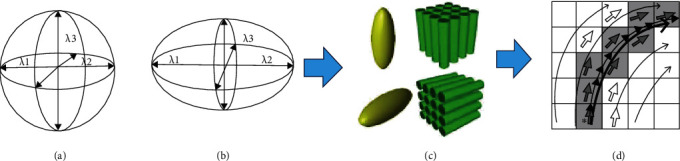
Biophysical presentation of axon fiber in DTI image.

When the 3 eigenvalues (*λ*1, *λ*2, and *λ*3) are equal, it means isotropic, as shown in Figure 2(a). Or if the 3 eigenvalues are not qual, it means anisotropic, as shown in Figure 2(b). The human or animal brain has its natural structure with axons shaped like fiber bundles, as shown in Figure 2(c). Therefore, the fiber orientations of neuron cells could be represented by three eigenvalues, as shown in Figure 2(d).

To better express the axon fiber alignment characteristics, the mean diffusion degree MD, diffusion anisotropy FA, axial diffusion coefficient AD, and radial diffusion coefficient RD are often used to represent the spatial changes of axon cells in each direction with the equations below:

Mean diffusivity (MD):

(2) $$\begin{array}{c} \text{MD}=\frac{\lambda_{1}+\lambda_{2}+\lambda_{3}}{3}. \end{array}$$

Fractional anisotropy (FA):

(3) $$\begin{array}{c} \text{FA}=\sqrt{\frac{3}{2}}\sqrt{\frac{{\left(\lambda_{1}-D\right)}^{2}+{\left(\lambda_{2}-D\right)}^{2}+{\left(\lambda_{3}-D\right)}^{2}}{\lambda_{1}^{2}+\lambda_{2}^{2}+\lambda_{3}^{2}}}. \end{array}$$

Axial diffusivity (AD):

(4) $$\begin{array}{c} \text{AD}=\lambda_{1}. \end{array}$$

Radial diffusivity (RD):

(5) $$\begin{array}{c} \text{RD}=\frac{\lambda_{2}+\lambda_{3}}{2}. \end{array}$$

### 2.4. Diffusion Tensor Imaging

In preprocessing and calculation of DTI parameters: FSL (Functional Magnetic Resonance Imaging of the Brain Software Library) toolbox in MATLAB 2018b was adopted to preprocess the original data and perform the tensor calculation. Firstly, all DTI images were adjusted by using nondiffusion weighted (*b* = 0) images as reference (including eddy current artifacts and motion artifacts). Then, the diffusion tensor eigenvalues (i.e., *λ*1, *λ*2, and *λ*3), mean diffusion coefficient (MD), and anisotropy fraction (FA) of each voxel were calculated based on all DTI images, and the corresponding images were generated through the FSL toolbox.

Then, these generated images are matched to the ICBM-DTI-81 (the International Consortium of Brain Mapping) standard space template with affine registration and nonlinear registration. This standardized space is automatically segmented white matter and named by the white matter segmentation map (WMPM). With the standard space template, the regions of interest (ROI) can be chosen conveniently while the DTI parameters of each ROI are also ready for statistically analyzed.

The SPSS statistical software was used to compare the four DTI parameters of each ROI between the mTBI group and the healthy control group. When the quantitative data met the normal distribution and homogeneous equation, two-sample independent*T*-test was adopted in our study; otherwise, the Mann–Whitney *U* test was used in the statistical analysis. When *P* is less than 0.05, the difference was considered to be statistically significant.

### 2.5. Correlation Analysis between Neuroimage and Neuropsychological Assessment

The correlation analysis between neuroimage and neuropsychological assessment was conducted including the Wechsler intelligence scale for verbal intelligence (e.g., knowledge and digit span) and operating intelligence (e.g., mapping and digit symbols) and the stroop color word test measuring cognitive-perform functions. The Pearson correlation coefficient analysis was measured through the correlation coefficient (*P* and *R*) with the algorithm shown in equations (6) and (7) using MATLAB. In equations (6) and (7), variable *A* represents DTI parameters (FA, MD, RD, and AD); variable *B* represents behavioral parameters (digit symbol, stroop color-word, digit span, and intelligence value); *μA* (or *μB*) and *σA* (*σB*) were the mean value and standard deviation of variable *A* (or *B*).

(6) $$\begin{array}{c} P\left(A,B\right)=\frac{1}{N-1}\overset{N}{\underset{i=1}{\sum }}\left(\frac{A_{i}-\mu A}{\sigma A}\right)\left(\frac{B_{i}-\mu B}{\sigma B}\right), \end{array}$$

(7) $$\begin{array}{c} R=\left(\begin{array}{cc} 1 & P\left(A,B\right)\\ P\left(B,A\right) & 1 \end{array}\right). \end{array}$$

After calculation, the correlation coefficients of the two variables were presented as the *R* and *P*. When the *R* value is close to 1, the two groups of variables have perfect positive correlation. The *R* value is close to -1; the two groups of variables have perfect negative correlation. The confidence space is reached when *P* is less than 0.05 [12].

## 3. Result

### 3.1. mTBI Patients' Neuropsychological Assessment

Compared to the control group, the digit symbol substitution test (DSST) performed significantly worse in the mTBI patients' group (*P* < 0.05). There was no significant statistical difference between the mTBI group and the control group in other neuropsychological tests, such as digit span (forward and reverse) and stroop color-word interference test (color-word interference time), as shown in Table 1.

### 3.2. DTI and Structure Network Comparisons between the mTBI Acute Patients and Control Group

**Figure 3:**
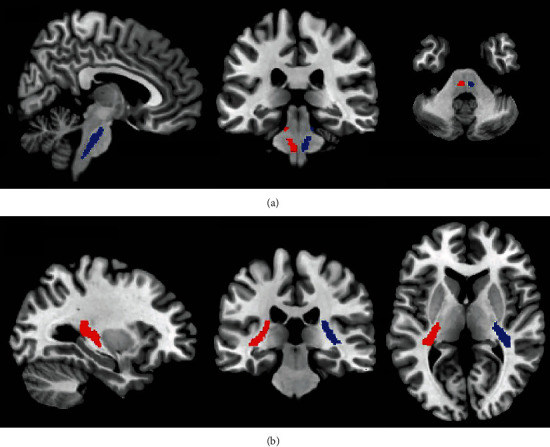
The mTBI patient brain region location with the significant AD value difference compared to the control group. Compared to the control group, the mTBI patient AD value increased in the following brain regions: (a) blue: left corticospinal tract and red: right corticospinal tract; (b) blue: right posterior internal capsule lenticula and red: left posterior internal capsule lenticula.

**Figure 4:**
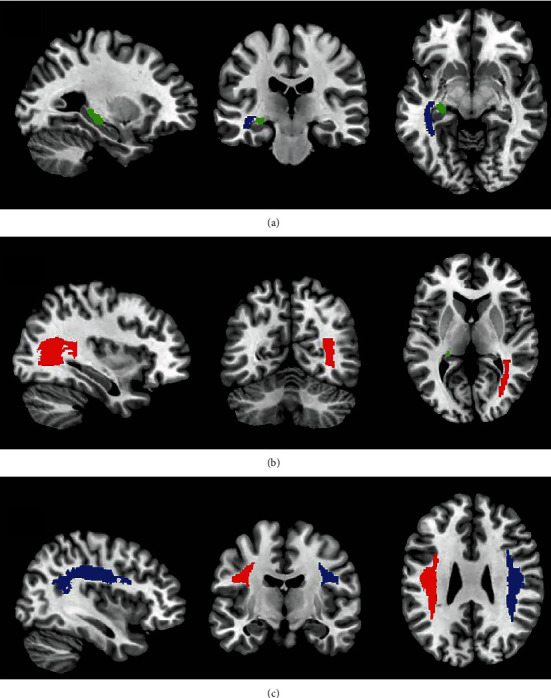
Brain region location with abnormal AD value in mTBI patients. Compared to the control group, the mTBI patient AD value increased in the following brain regions: (a) blue: left sagittal plane and green: terminal striae; (b) red: left posterior thalamic radiation and green: terminal striatum; (c) blue: upper right longitudinal fiber and red: upper left longitudinal fiber.

**Figure 5:**
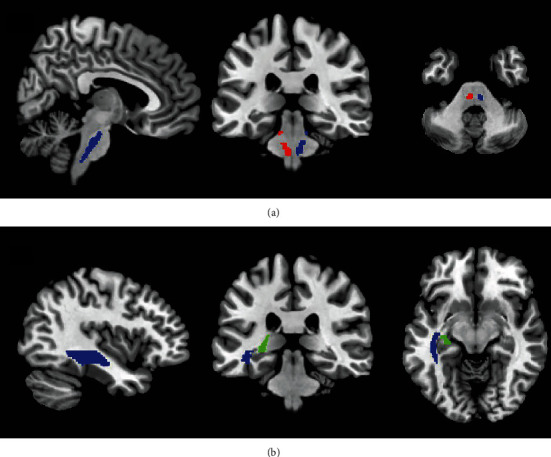
Brain region location with abnormal MD value in mTBI patients. Compared to the control group, the mTBI patient MD value increased in the following brain regions: (a) blue: left corticospinal tract and red: right corticospinal tract; (b) blue: right posterior internal capsule lenticula and red: left posterior internal capsule lenticula. Compared to the control group, the mTBI patient MD value increased in the following brain regions: (a) blue: left corticospinal tract and red: right corticospinal tract; (b) blue: sagittal plane and green: terminal striae.

After calculation and statistical analysis, we found that DTI parameters in the acute stage of mTBI patients have varying degrees compared to the healthy control group. No significant statistical difference was found in FA and RD values. The axial diffusion coefficient AD increased significantly (*P* < 0.05) in the following brain regions: bilateral corticospinal tracts (CST), bilateral posterior internal capsule lenticula, bilateral superior longitudinal fasciculus, left terminal striae and left sagittal plane (including inferior longitudinal fasciculus and inferior fronto-occipital fasciculus), and right posterior thalamic radiation (shown in Figures 3 and 4). The mean diffusivity (MD) in the bilateral corticospinal tract, left fornix, and left sagittal plane was significantly higher than that in the healthy control group (*P* < 0.05), as shown in Figure 5.

### 3.3. Correlation Analysis between Neuroimage and Neuropsychological Assessment

**Figure 6:**
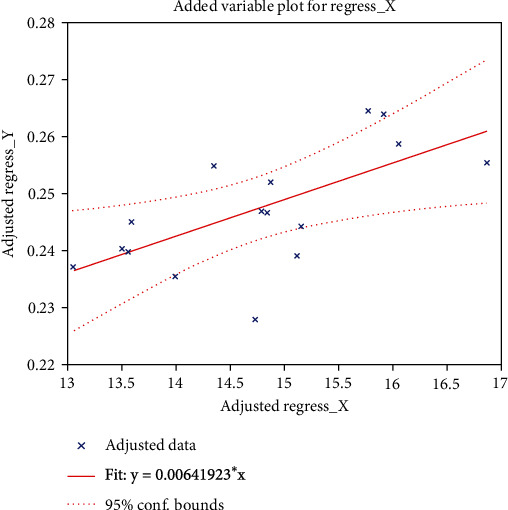
Correlation analysis between cognitive psychology assessment DSST and the AD values. *X*-axis represents the score of digital symbol substitution experiment (DSST), and *Y*-axis represents the AD value in left sagittal plane.

**Figure 7:**
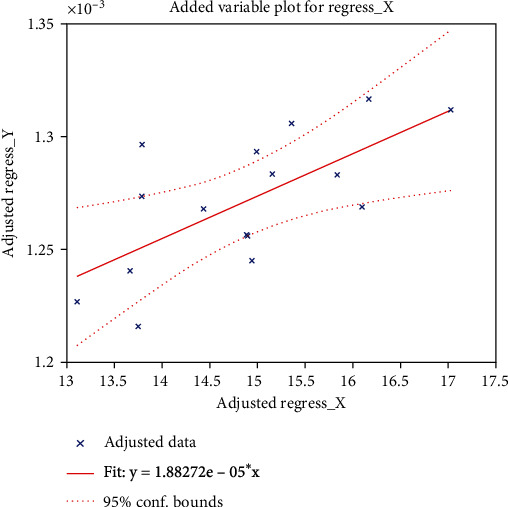
Correlation analysis between cognitive psychology assessment DSST and the MD values. *X*-axis represents the score of digital symbol substitution experiment (DSST), and *Y*-axis represents the MD value in left sagittal plane.

**Figure 8:**
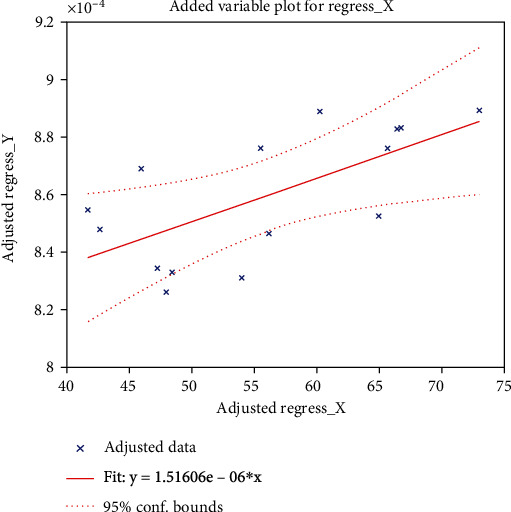
Correlation analysis between cognitive psychology assessment digit span and the AD values. *X*-axis represents the score of digital spans, and *Y*-axis represents the AD value in left sagittal plane.

The Pearson correlation coefficients were calculated using DSTT and digit span (which were significantly different between the patient and control groups among 14 neuropsychological assessments) as well as MD and AD. We found that DSTT performance was associated with the increased AD value in the left sagittal plane (*R* = 0.526; *P* = 0.012), as shown in Figure 6. Results of DSST neuropsychological assessments and the elevation of MD value in the left sagittal plane were positively correlated in acute stage mTBI patients (*R* = 0.331; *P* = 0.0255), as shown in Figure 7. There was also a positive correlation between digital span assessment and the increased AD value in the left sagittal plane in acute mTBI patients (*R* = 0.506; *P* = 0.017), as shown in Figure 8.

## 4. Discussion

In this study, we intend to locate the structurally different brain regions in mTBI acute stage patients and analyze the correlation between these brain regions and neuropsychological change after mTBI. Fourteen neuropsychological assessments were performed on 77 mTBI acute stage patients and 72 healthy control participants, combined with a multiparameter analysis of DTI. Compared to the healthy control group, the patients in the acute stage of mTBI presented lower scores in the DSST, suggesting that mTBI patients in the acute stage had a decline in information processing speed and associative learning. The difference in DTI parameters in acute stage mTBI patients was mainly manifested with increased AD and MD values in multiple brain regions, while RD and FA values showed no significant difference. The most significant differences were found in bilateral CST, bilateral posterior internal capsule lentiform nucleus, bilateral superior longitudinal fasciculus, left terminal striae and left sagittal plane (including inferior longitudinal fasciculus and inferior fronto-occipital fasciculus), and right posterior thalamic radiation. The significantly positive Pearson correlation was found between AD and MD elevation in the left sagittal layer and between the results of DSST and digit span in acute stage mTBI patients.

Narayana et al. [13] and Henry et al. [14] both reported the increased AD values in multiple brain regions of acute stage mTBI patients, including the bilateral corticospinal tracts (CST) as found in our study. These findings were consistent with our findings, but they did not perform neuropsychological assessments. In the acute stage of mTBI, the glial cell proliferation was reported to be associated with the increased AD value, which could be pathologically represented by the incomplete rupture of axon cells during the acute phase of mTBI [15].

As reported in pathological studies of acute phase mTBI, the axial membrane calcium channel permeability increased and Ca2+ influx was induced when the axon was stretched but not completely broken under mechanical force or acceleration [16]. Anatomically, the superior longitudinal tract is located on the dorsal and lateral of the radiative crown and links to the four white matter fiber bundles (the frontal, parietal, occipital, and temporal cortices) as a bridge. The parietal lobe white matter contains the superior longitudinal tract, and the white matter of the temporal lobe contains the inferior longitudinal tract, which primarily connects the occipital lobe to the temporal cortex. This special and key anatomical structure determined that the injury of the upper and lower longitudinal tracts will influence the speed of information processing and learning ability. Spitz et al. [17] reported that the FA values of the right upper and lower longitudinal tracts in the TBI patients were significantly correlated with DSST performance in the 68 chronic phase TBI patients (the severity of TBI ranged from mild to severe). Previous studies indicated that the significant different structure of upper longitudinal white matter could lead to a decrease in information processing speed in cerebral infarction patients [18, 19]. Mazerolle et al. [20] found that the FA value of upper longitudinal descent was correlated with the decline of information processing speed in multiple sclerosis patients. Based on these previous studies and our findings, we suggest that the diagnosis and treatment of mTBI patients in the acute phase should pay more attention to the abnormal structures of the upper longitudinal bundles of white matter and the patients' information processing speed.

Our study found no significant difference in RD value between acute stage mTBI patients and the control group. According to previous studies, RD value is mainly related to demyelination while the acute phase mTBI is more likely to suffer axon swelling. The demyelinating lesions are more possible to happen in the chronic phase of mTBI. Another factor might be due to that the traumatic brain injuries are mild and the cytotoxic edema is not often serious in mTBIs. Therefore, there was no significant change in RD values. In this study, the MD value was treated as the average diffusion rate of the three directions. When the AD value of axial diffusion increases and the RD value remains unchanged, the MD value will increase with the AD value.

There are some limitations in this study. Firstly, most of the subjects were middle-aged and young with the age of 18-40 years; children and elderly are not leave out of consideration. Although this criterion can eliminate the interference factors caused by age and brain development, the data from the subjects of more varying ages might bring additional knowledge on the performance of neuroimage biomarkers in mTBI patients. Secondly, the mTBI patients were not divided into groups according to the mechanical loading features leading to mTBIs. From the perspective of mechanical mechanisms, the mechanical loading location and directions to the head may be highly associated to the location of the injured brain regions. If there were more than 200 samples for instance, collision location on the head (e.g., frontal impact, lateral impact, and occipital impact) could be considered as a grouping criterion.. Thirdly, multiple follow-ups could be implemented within 2 weeks. For example, DTI scan and cognitive assessments can be performed on the first day, the third day, the seventh day, and the second week after injury, which will be helpful to observe the dynamic change in DTI parameters and neurophysiological changes.

## 5. Conclusion

The acute phase mTBI patients showed lower scores on DSST than those in the healthy control group. This neuropsychological change was associated with the increased AD value and MD value in the left sagittal layer, which indicated a reduction of information processing speed in mTBI patients in the acute phase. It might be related to abnormal AD value and MD value in the upper longitudinal tract, lower longitudinal tract, lower frontal occipital tract, and sagittal layer. In this study, combined with neuropsychological test and an increase of the AD value and MD value in certain brain regions, surgeons should pay more attention to the abnormal structures of the upper longitudinal tract and the patients' information processing speed in the diagnosis and treatment of the acute phase mTBI patients.

## Figures and Tables

**Table 1:** Statistical analysis of cognitive behavior test.

Cognitive behavior test	Mean value ± SD		*P* value
	mTBI group	Control group	
Age	28.41 ± 9.36 (Y)	28.55 ± 8.74 (Y)	0.96
IQ	102.35 ± 15.67	105.67 ± 11.98	0.48
Knowledge	18.97 ± 5.02	16.76 ± 5.78	0.132
Knowledge standard score	11.17 ± 2.72	10.03 ± 3.24	0.152
Digit span	14.25 ± 2.27	13.68 ± 3.11	0.228
Digit span standard score	12.75 ± 2.79	11.76 ± 3.29	0.128
Forward	8.31 ± 0.92	7.89 ± 1.65	0.193
Reverse	6.17 ± 1.48	5.54 ± 1.66	0.063
Picture completion	11.39 ± 2.83	11.24 ± 3.04	0.705
Picture completion standard score	8.86 ± 2.17	8.78 ± 2.51	0.809
Digit symbol	56.27 ± 10.27	62.35 ± 12.79	0.026
Stroop_word	23.75 ± 5.45	22.39 ± 6.81	0.088
Stroop_color	36.61 ± 5.30	36.47 ± 11.04	0.168
Stroop_D	60.70 ± 14.46	63.59 ± 21.47	0.873
Stroop_D-C	24.10 ± 12.36	27.13 ± 13.91	0.663

## Data Availability

The raw data supporting the conclusions of this article will be made available by the authors, without undue reservation.
